# TRPV1 Channels and Gastric Vagal Afferent Signalling in Lean and High Fat Diet Induced Obese Mice

**DOI:** 10.1371/journal.pone.0135892

**Published:** 2015-08-18

**Authors:** Stephen J. Kentish, Claudine L. Frisby, Stamatiki Kritas, Hui Li, George Hatzinikolas, Tracey A. O’Donnell, Gary A. Wittert, Amanda J. Page

**Affiliations:** 1 Centre for Nutrition and Gastrointestinal Diseases, Discipline of Medicine, University of Adelaide, Adelaide, South Australia, Australia; 2 South Australian Health and Medical Research Institute (SAHMRI), Adelaide, South Australia, Australia; 3 Royal Adelaide Hospital, Adelaide, South Australia, Australia; 4 Women’s & Children’s Hospital, Adelaide, South Australia, Australia; INSERM, FRANCE

## Abstract

**Aim:**

Within the gastrointestinal tract vagal afferents play a role in control of food intake and satiety signalling. Activation of mechanosensitive gastric vagal afferents induces satiety. However, gastric vagal afferent responses to mechanical stretch are reduced in high fat diet mice. Transient receptor potential vanilloid 1 channels (TRPV1) are expressed in vagal afferents and knockout of TRPV1 reduces gastro-oesophageal vagal afferent responses to stretch. We aimed to determine the role of TRPV1 on gastric vagal afferent mechanosensitivity and food intake in lean and HFD-induced obese mice.

**Methods:**

TRPV1+/+ and -/- mice were fed either a standard laboratory diet or high fat diet for 20wks. Gastric emptying of a solid meal and gastric vagal afferent mechanosensitivity was determined.

**Results:**

Gastric emptying was delayed in high fat diet mice but there was no difference between TRPV1+/+ and -/- mice on either diet. TRPV1 mRNA expression in whole nodose ganglia of TRPV1+/+ mice was similar in both dietary groups. The TRPV1 agonist N-oleoyldopamine potentiated the response of tension receptors in standard laboratory diet but not high fat diet mice. Food intake was greater in the standard laboratory diet TRPV1-/- compared to TRPV1+/+ mice. This was associated with reduced response of tension receptors to stretch in standard laboratory diet TRPV1-/- mice. Tension receptor responses to stretch were decreased in high fat diet compared to standard laboratory diet TRPV1+/+ mice; an effect not observed in TRPV1-/- mice. Disruption of TRPV1 had no effect on the response of mucosal receptors to mucosal stroking in mice on either diet.

**Conclusion:**

TRPV1 channels selectively modulate gastric vagal afferent tension receptor mechanosensitivity and may mediate the reduction in gastric vagal afferent mechanosensitivity in high fat diet-induced obesity.

## Introduction

Transient receptor potential (TRP) channels comprise a superfamily of nonselective cation channels [1]. Initially described as “Thermo TRPs” [2], more recent investigations have shown that they respond to a number of different stimuli. For example, it has been demonstrated that several TRP vanilloid (V) channels, including TRPV1, are also mechano- and osmo-sensitive [3]. The TRPV1 channel is best known as a mediator of noxious or painful stimuli [4]. However, since its discovery, physiological roles of TRPV1, not associated with pain are becoming apparent, including roles in the regulation of energy balance and intermediary metabolism [5, 6]. It has been suggested that activation of TRPV1 channels might be a target for the management of obesity [7].

Gastrointestinal (GI) vagal afferent nerves play an important role in the control of food intake and satiety signalling. An important component of these satiety signals involves gastric vagal afferents which respond to mechanical distension of the stomach [8, 9]. Within the wall of the stomach there are two classes of mechanically sensitive gastric vagal afferents. As mentioned, in the muscular layer, tension receptors detect fullness by responding to distension and contraction of the stomach wall [8, 10]. In contrast, mucosal receptors are excited by mechanical forces associated with contact of larger food particles with the epithelium, and thus are likely to be involved in the discrimination of particle size. The net effect of activating mucosal afferents is to trigger vagal reflexes which slow gastric emptying and facilitate mechanical digestion in the stomach [11]. In high fat diet (HFD) induced obesity there is a dampened response of gastric tension receptors to stretch [12] which may contribute to the facilitation of ongoing food intake despite energy sufficiency and an expanded adipose tissue store.

TRPV1 channels are expressed in the nodose ganglia [13] and more specifically in cell bodies of vagal afferents innervating the upper GI tract [14]. Gastro-oesophageal vagal afferent responses to distension are attenuated in TRPV1-/- mice [15] indicating a role in gastric tension receptor mechanosensitivity. It is plausible that the HFD induced reduction in gastric vagal afferent mechanosensitivity is due to changes in TRPV1 mediated signalling. Therefore we aimed to determine whether this was the case, by using an *in vitro* gastro-oesophageal vagal afferent preparation from TRPV1 intact and deficient mice fed either a standard laboratory or high fat diet.

## Materials and Methods

All studies were approved and performed in accordance with the guidelines of the Animal Ethics Committees of the University of Adelaide and Institute for Medical and Veterinary Science, Adelaide, Australia. Every attempt was made to limit the number of animals used and minimize their suffering.

### Animals

All mice in these studies were bred from Jackson Laboratories (Bar Harbour, ME, USA) C57BL/6J (TRPV1+/+) and B6.129X1-TRPV1^tm1Jul^/J (TRPV1-/-) mice. 20 male TRPV1+/+ and 20 male TRPV1-/- mice were individually housed under a 12 hour light: dark cycle (lights on at 06:00) with free access to food and water. After a week to acclimatize 8 week old mice were assigned randomly to either a 20 week standard laboratory diet (SLD) group (Energy: 12% from fat, 65% from carbohydrate and 23% from protein; Specialty feeds, Glen Forest, Western Australia, Standard meat free rat and mouse diet; N = 20 (N = 10 TRPV1+/+ and N = 10 TRPV1-/-)) or a 20 week HFD group (Energy: 60% from fat, 20% from carbohydrate and 20% from protein; Research diets Inc., New Brunswick, USA, #D12492); N = 20 (N = 10 TRPV1+/+ and N = 10 TRPV1-/-)). The mice were weighed weekly and food intake monitored over the 20 week diet period. Gonadal fat pad mass and stomach content were determined from all mice on the day they were used for the *ex-vivo* mouse gastro-oesophageal vagal afferent preparation. A separate cohort of male C57Bl/6J mice (N = 12) were assigned either a SLD (N = 6) or a HFD (N = 6) for 20 weeks prior to electrophysiology studies to investigate the effect of a TRPV1 agonist, N-oleoyldopamine (OLDA), on gastric vagal afferent mechanosensitivity.

### Breath test analysis to determine gastric emptying rate

The effect of diet and disruption of TRPV1 channels on gastric half emptying time of a solid meal (t ½) was determined at the 18 week time point using breath test analysis as previously described [16, 17]. Briefly, mice were fasted overnight. Following the baseline breath sample collection, mice were given 0.1 g of baked egg yolk containing 1 μg ml^-1^ of ^13^C-labelled octanoic acid (99% enrichment, Cambridge Isotope Laboratories) to consume within 1min. Breath samples were collected at regular intervals (0–150 min) and analysed, with an isotope ratio mass spectrometer (ABCA 20/20 Europa Scientific, Crewe, UK), for ^13^CO_2_ content. Nonlinear regression analysis for curve fitting was used to calculate gastric half-emptying time (t ½).

### *In vitro* mouse gastro-oesophageal afferent preparation

This preparation has been described in detail previously [18, 19]. In short, TRPV1+/+ or TRPV1-/- male mice on the SLD or HFD diets were anaesthetised with isoflurane and sacrificed via exsanguination between 0900 and 0930hr. The stomach and oesophagus, with intact vagal nerves, were removed and placed mucosa side up in an organ bath containing a modified Krebs solution comprised of (in mM): 118.1 NaCl, 4.7 KCl, 25.1 NaHCO_3_, 1.3 NaH_2_PO_4_, 1.2 MgSO_4_.7H_2_O, 1.5 CaCl_2_, 1.0 citric acid, 11.1 glucose and 0.001 nifedipine, bubbled with 95% O_2_- 5% CO_2_.

### Characterization of gastric vagal afferents

In mice two types of mechanosensitive gastric vagal afferent have been reported [18]: mucosal receptors respond to mucosal stroking, but not to circular tension and tension receptors respond to both mucosal stroking and circular tension. Mucosal stroking was performed using calibrated von Frey hairs (10–1000mg) stroked across (5mms^-1^) the receptive field. The mechanical response of the middle eight of ten strokes was taken for analysis. Circular tension was applied using a threaded hook attached to a cantilever system via a pulley close to the preparation. Weights (1–5g) were placed on the opposite end of the cantilever for one minute. A break of at least a minute was allowed between removing one weight and applying the next.

### Effect of N-oleoyldopamine on the mechanosensitivity of gastric vagal afferents

After mechanical sensitivity of the gastric vagal afferents had been established, the effect of the TRPV1 agonist N-oleoyldopamine (OLDA) on mechanical sensitivity was determined. OLDA (0.1μM) was added to a ring placed over the receptive field and allowed to equilibrate for 5 min after which time the ring was removed and the responses to 3g circumferential stretch for tension receptors and the stroke-response (10–1000mg) curves for mucosal receptors were redetermined. This procedure was repeated for OLDA at increasingly higher doses (1–10 μM). Time-controlled experiments were performed in which there was no significant change in the mechanical responses over a comparable duration.

### Data recording

Afferent impulses were amplified with a biological amplifier (DAM 50, World Precision Instruments, Sarasota, FL, USA), and filtered (band-pass filter 932, CWE, Ardmore, PA, USA). Single units were discriminated on the basis of action potential shape, duration, and amplitude by use of Spike 2 software (Cambridge Electronic Design, Cambridge, UK).

### Nodose ganglia quantitative RT-PCR

Nodose ganglia were removed bilaterally from TRPV1+/+ mice on both diets. Total RNA was extracted using an Invitrogen PureLink RNA Micro Kit (Life Technologies, Mulgrave, Australia), according to the manufacturer’s instructions, and included an on-column DNase step for DNA-free RNA. Total RNA yield and quality were quantified on a NanoDrop Lite spectrophotometer (Thermo Fisher Scientific, Scoresby, Australia) with an OD_260/280_ of greater than 1.8 accepted as sufficiently pure for downstream amplification. cDNA synthesis from total RNA was performed using the Superscript III First-Strand Synthesis Supermix Kit (Cat. No.18080-400, Life Technologies) according to manufacturer’s instructions. Taqman Fast Advanced Master Mix (Cat. No. 4444964, Life Technologies) and pre-designed, pre-formulated Taqman Gene Expression Assays with FAM dye labelled MGB probe which targeted protein-coding transcripts for TRPV1 (Assay ID: Mm01246302_m1) and beta-2-microglobulin (B2m, Assay ID: Mm00437762_m1) were used to perform the real-time RT-PCR reactions on a 7500 Fast Real-time PCR System (Life Technologies). The control assay, B2m, was chosen to normalise the amount of cDNA added for each sample as it did not vary significantly between diet groups. No template controls, for each gene expression assay, were performed in duplicate and four technical replicates were run for each reaction. For each individual PCR reaction the mix contained; Taqman Fast Advanced Master Mix 5μL, cDNA template 2.5μL (5ng), 0.5μL Taqman Gene Expression Assay and 2μL nuclease-free water to make a total volume of 10μL. Thermal-cycling profile included; UNG incubation 2min 50°C, polymerase activation 20sec 95°C, PCR (40 cycles) 0.3sec 95°C (Denature) and 30sec 60°C (Anneal/extend).

Data were analysed using the 2^-∆CT^ method with DataAssist^TM^Real Time PCR Analysis Software (Life Technologies).

### Statistical analysis

All data in graphs are expressed as mean values ± SEM with N = number of animals studied. Vagal afferent stimulus-response curves and weight change were analysed using two-way analysis of variance (2-way ANOVA) and Bonferroni post hoc tests. RNA levels, fat mass, food intake and gastric emptying rate were analysed using a one-way ANOVA with a Tukey post hoc test. Significance was defined at p<0.05.

## Results

### Diet induced changes to mouse weight and fat mass

**Fig 1:**
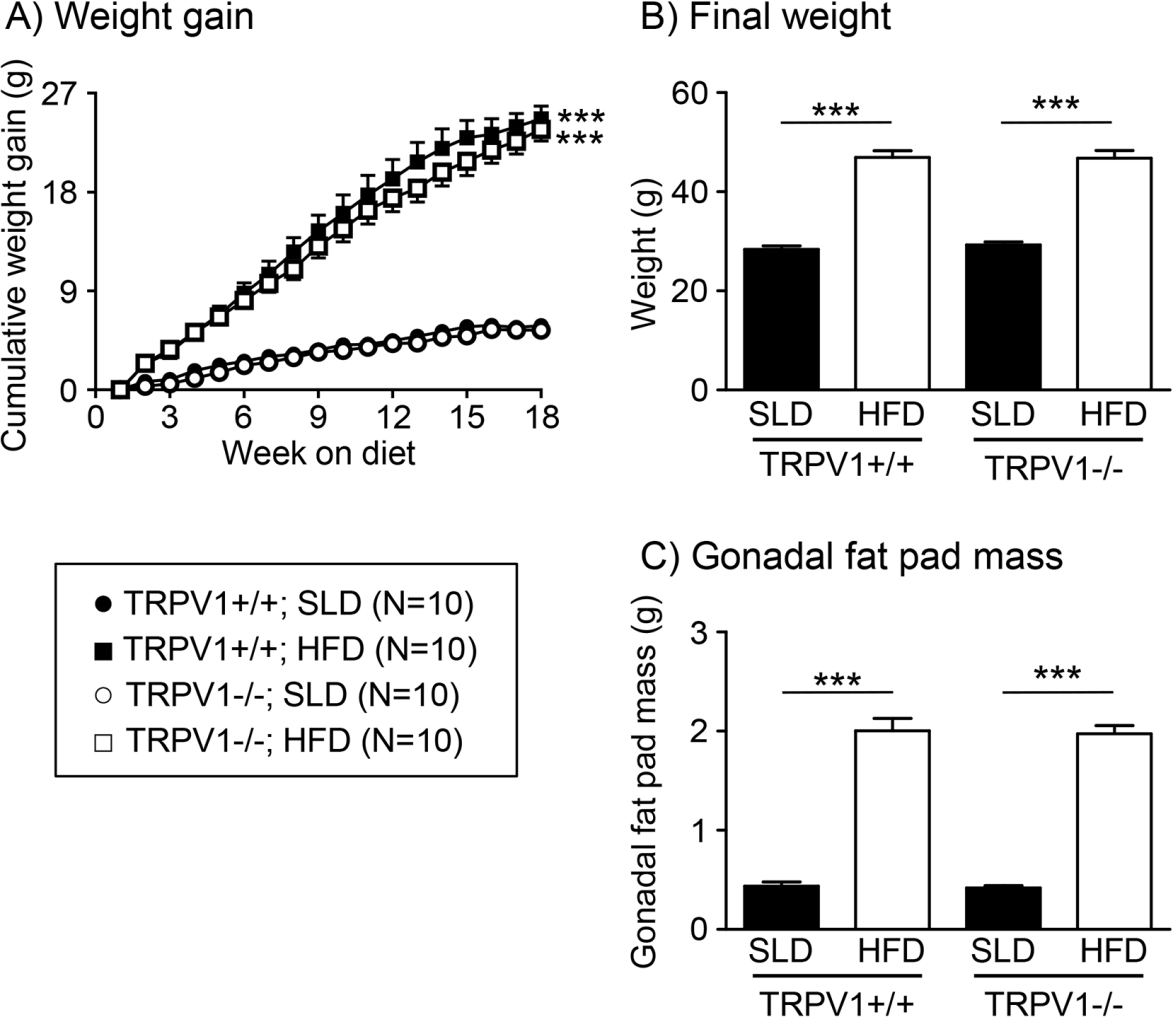
Diet dependent changes to mouse body parameters. (A) The weight gain of TRPV1+/+ (solid symbols) and TRPV1-/- (open symbols) mice fed either a standard laboratory diet (SLD, N = 10/group; ● and ○) or a high fat diet (HFD, N = 10/group; ■ and □). The final body weight (B) and gonadal fat pad mass (C) of TRPV1+/+ and-/- mice fed a SLD (solid bars) or HFD (open bars) at the end of the 20 week diet regime., ***p<0.001 vs. SLD

The HFD mice gained more weight than the SLD mice (Fig 1A and 1B; p<0.001 in both TRPV1+/+ and-/- mice). There was no difference in weight between TRPV1+/+ and-/- mice on either diet (Fig 1A and 1B). The gonadal fat pad mass was significantly higher in mice fed a HFD compared to mice fed a SLD (Fig 1C; p<0.001 in both TRPV1+/+ and-/- mice). There was no difference in gonadal fat pad mass between TRPV1+/+ and-/- mice on either diet (Fig 1C).

### High fat diet induced changes in gastric mechanosensitivity are not observed in TRPV1-/- mice

**Fig 2:**
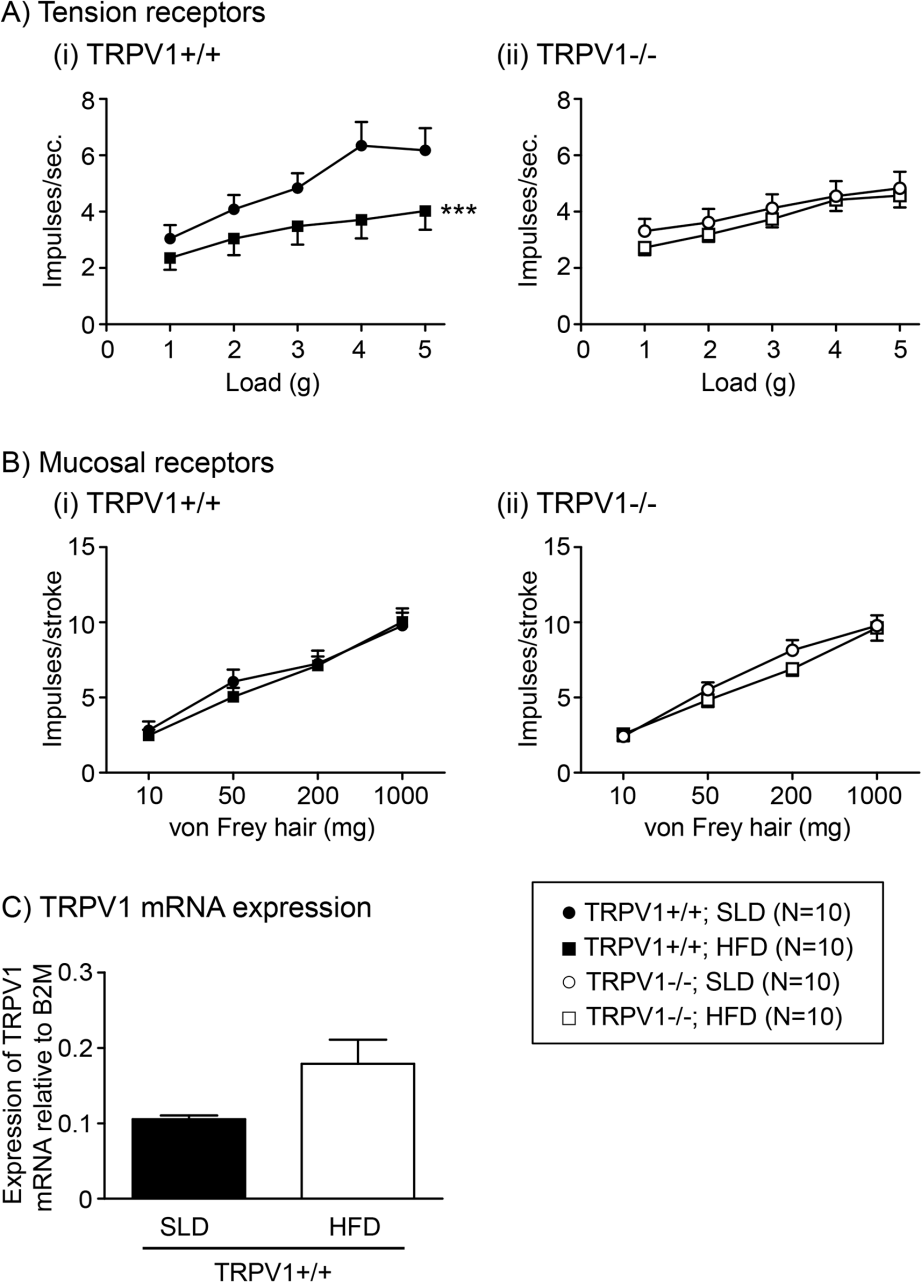
Mechanosensitivity of gastric vagal afferents in TRPV1+/+ and-/- mice. Stimulus response functions of tension-sensitive (A) and mucosal (B) gastric vagal afferents from (i) TRPV1+/+ and (ii) TRPV1-/- mice fed a SLD (● or ○, N = 10) or HFD (■ or □, N = 10). *** p<0.001 vs. SLD. (C) TRPV1 channel mRNA content in whole nodose ganglia of TRPV1+/+ mice fed a SLD (closed bar; N = 6) or HFD (open bar; N = 6).

Gastric tension receptor mechanosensitivity in TRPV1+/+ HFD mice is significantly reduced compared to the TRPV1+/+ SLD mice (Fig 2Ai, p<0.001). In contrast, there was no difference in the mechanosensitivity of TRPV1-/- mice fed either a SLD or HFD (Fig 2Aii). There was no difference in the response to mucosal stroking by mucosal receptors between any of the groups of mice (Fig 2Bi and ii).

TRPV1 mRNA content was observed in the whole nodose ganglia of TRPV1+/+ mice (Fig 2C). There was no significant difference in the level of TRPV1 mRNA content in the whole nodose ganglia of TRPV1+/+ mice fed either a SLD or HFD (Fig 2C).

**Fig 3:**
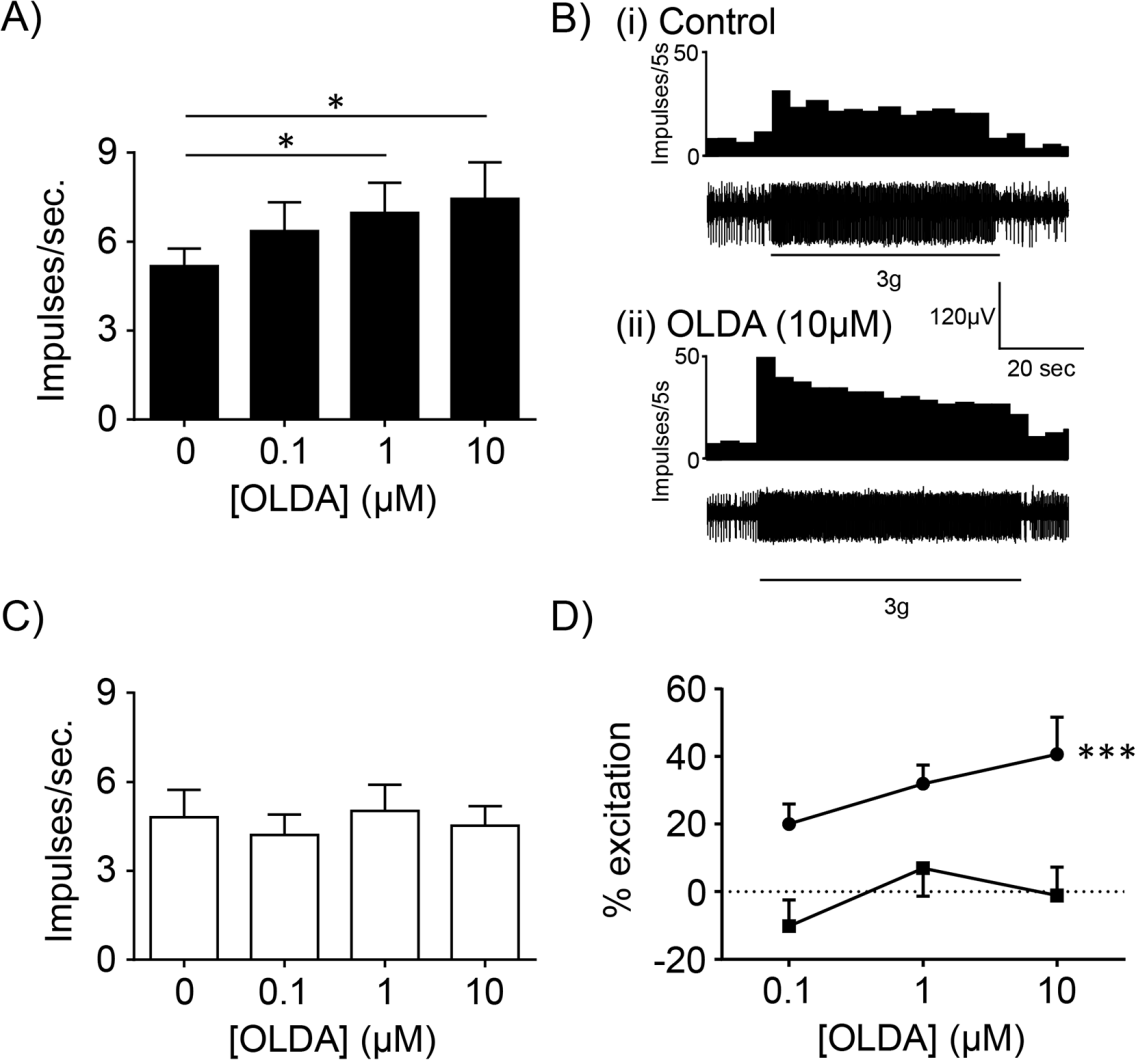
Mechanosensitivity of gastric vagal afferent tension receptors in the absence and presence of N-oleoyldopamine (OLDA). (A) In mice fed a SLD (N = 6), the response of gastric tension receptors to 3 g circular stretch. * p<0.05 vs. 0μM OLDA. (B) Typical response of a gastric tension receptor from a SLD mouse to 3 g tension in the (i) absence and (ii) presence of the TRPV1 agonist OLDA (10μM). (C) In mice fed a HFD (N = 6), the response of gastric tension receptors to 3 g circular stretch. (D) Percentage potentiation, in the presence of OLDA (0.1–10 μM), of gastric tension receptor responses to 3 g circular stretch in mice fed a SLD (■; N = 6) or a HFD (●: N = 6). ***p<0.001; diet effect, two-way ANOVA.

**Fig 4:**
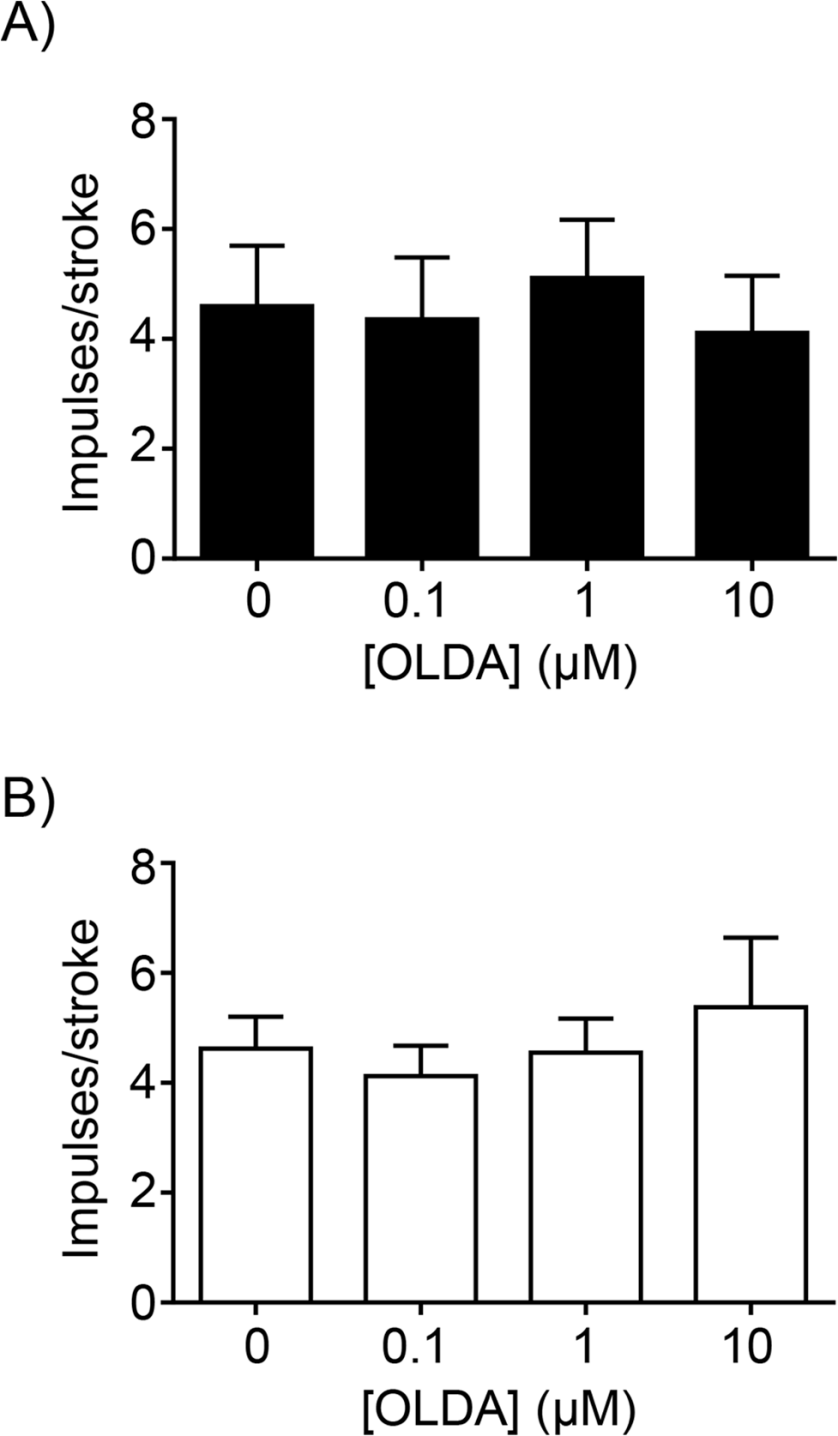
Mechanosensitivity of gastric vagal afferent mucosal receptors in the absence and presence of N-oleoyldopamine (OLDA). The response of gastric mucosal receptors to mucosal stroking with a 50mg von Frey hair in (A) mice fed a SLD (N = 4) and (B) mice fed a HFD (N = 5).

The TRPV1 agonist, OLDA (1–10μM), significantly increased the response of gastric tension receptors to 3g circular tension in mice fed a SLD (N = 6; Fig 3A & 3B) but not in mice fed a HFD (N = 6; Fig 3C). When the percentage potentiation was plotted against the concentration of OLDA it was evident that diet significantly altered the response to mechanical stimulation in the presence of OLDA (Fig 3D: p<0.001; diet effect, two-way ANOVA). OLDA (1–10μM) had no effect on the response of gastric mucosal receptors to stroking with a 50mg von Frey hair in mice fed a SLD or HFD (N≥4; Fig 4).

### Disruption of TRPV1 channels reduces the mechanosensitivity of gastric tension receptors and leads to associated changes in food intake in mice fed a SLD

**Fig 5:**
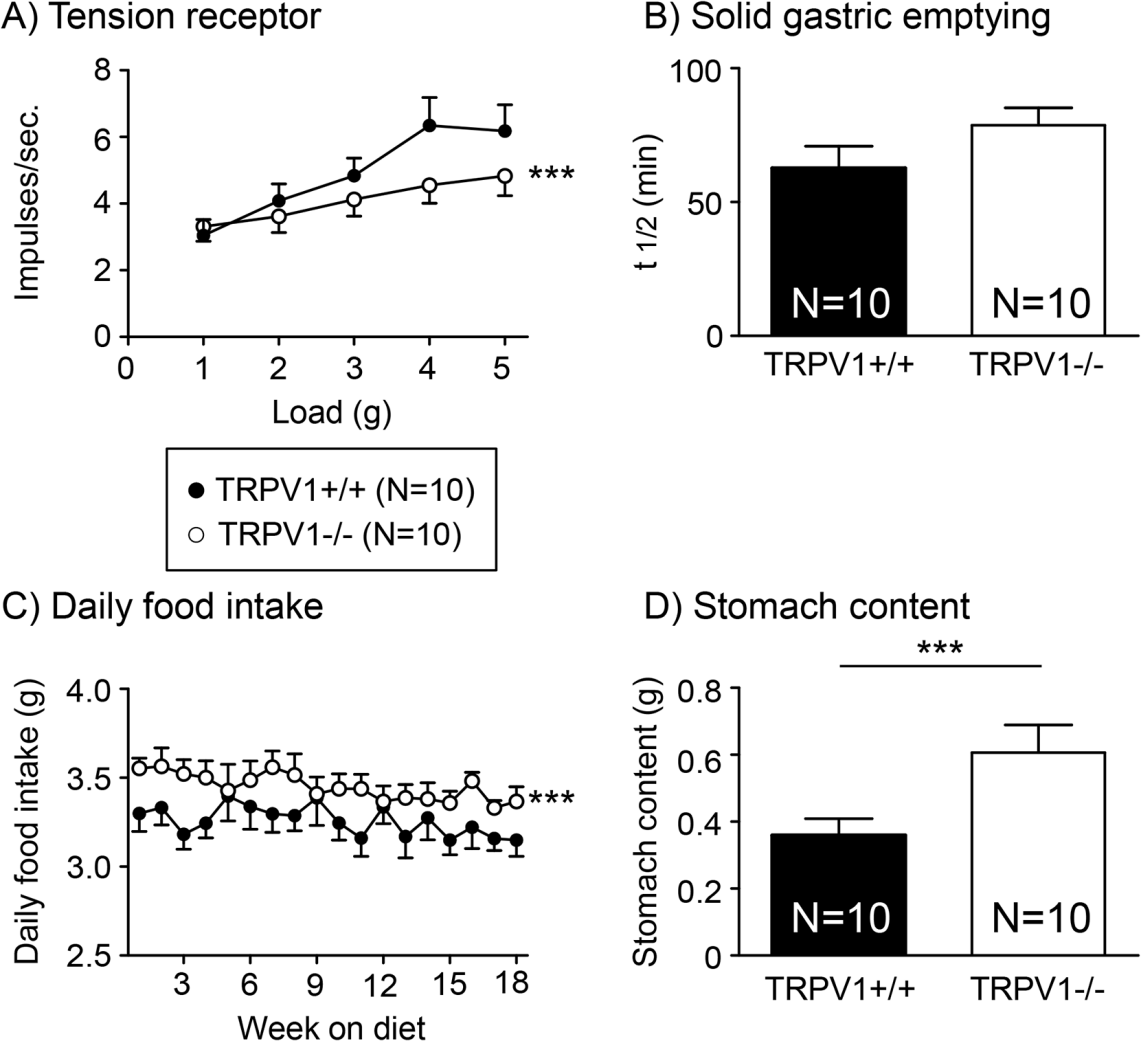
Tension receptor mechanosensitivity, food intake and gastric emptying in TRPV1+/+ and-/- SLD mice. (A) The response of gastric tension receptors to circular stretch (1–5g) in TRPV1+/+ (●) and TRPV1-/- (○) mice fed a SLD. (B) Gastric half emptying time (t1/2) of a solid meal in TRPV1+/+ (closed bar; N = 10) and TRPV1-/- (open bar; N = 10) mice fed a SLD. (C) Daily food intake in TRPV1+/+ (●; N = 10) and TRPV1-/- (○; N = 10) mice fed a SLD. (D) Stomach content at the end of the 20 week diet period at the point of collection of tissue for experimentation (0900–0930hr) in TRPV1+/+ (closed bar; N = 10) and TRPV1-/- (open bar; N = 10) mice fed a SLD. *** p<0.001 vs TRPV1+/+ mice.

Gastric tension receptor mechanosensitivity is significantly reduced in TRPV1-/- compared to TRPV1+/+ mice fed a SLD (Fig 5A; p<0.001). There was no difference in the rate of gastric emptying of a solid meal between TRPV1+/+ and-/- mice fed a SLD (Fig 5B). Consistent with the observed differences in gastric vagal afferent mechanosensitivity daily food intake was higher in the TRPV1-/- mice compared to the TRPV1+/+ mice fed a SLD (Fig 5C; p<0.001). In addition, stomach content at the end of the diet period was significantly greater in the TRPV1-/- mice compared to the TRPV1+/+ mice fed a SLD (Fig 5D; p<0.001).

### Disruption of the TRPV1 channel fails to affect the mechanosensitivity of gastric tension receptors in mice fed a HFD

**Fig 6:**
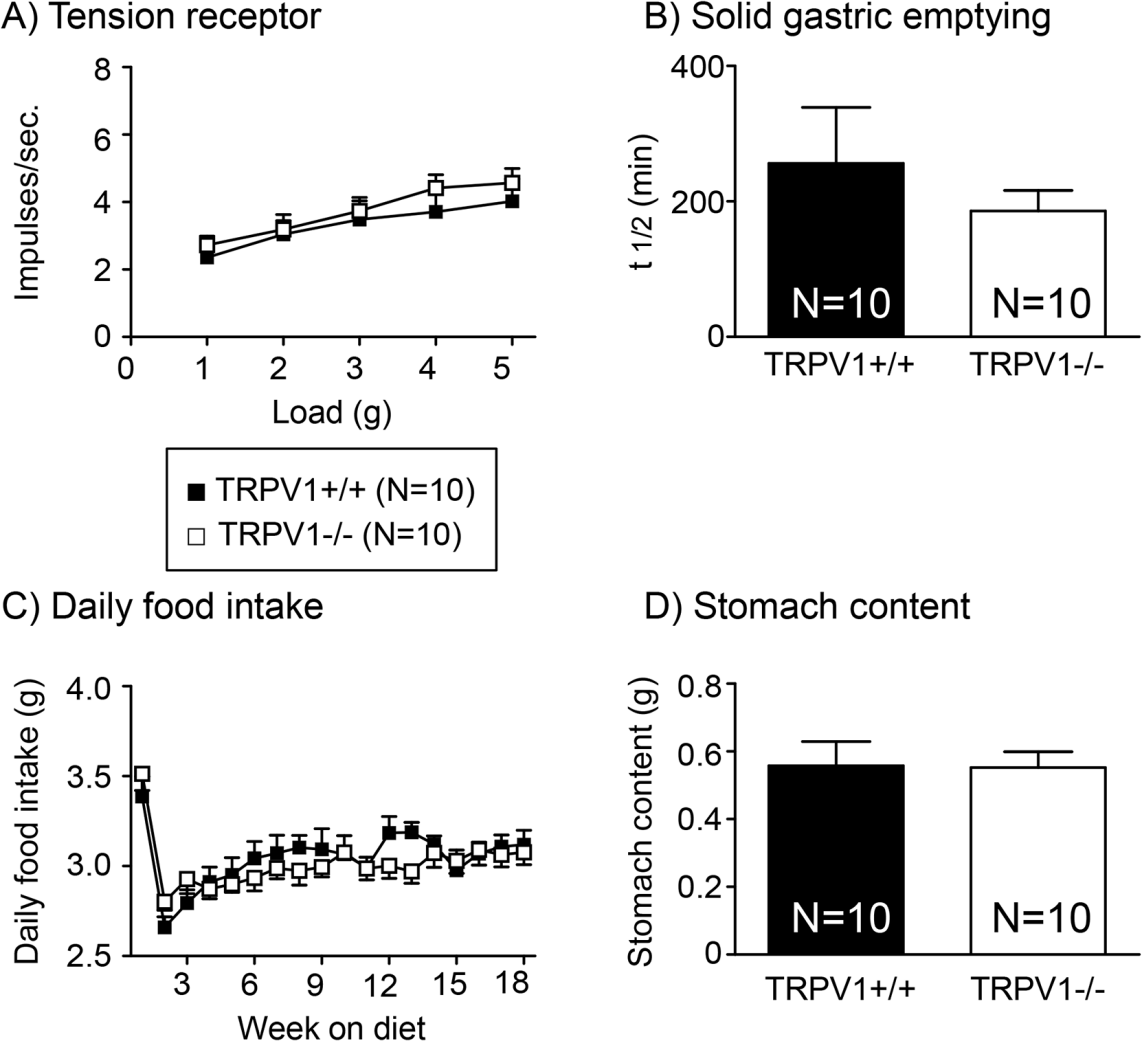
Tension receptor mechanosensitivity, food intake and gastric emptying in TRPV1+/+ and-/- HFD mice. (A) The response of gastric tension receptors to circular stretch (1–5g) in TRPV1+/+ (●) and TRPV1-/- (○) mice fed a HFD. (B) Gastric half emptying time (t1/2) of a solid meal in TRPV1+/+ (closed bar; N = 10) and TRPV1 -/- (open bar; N = 10) mice. (C) Daily food intake in TRPV1+/+ (●; N = 10) and TRPV1-/- (○; N = 10) mice fed a HFD. (D) Stomach content at the end of the 20 week diet period at the point of collection of tissue for experimentation (0900–0930hr) in TRPV1+/+ (N = 10) and TRPV1-/- (N = 10) mice fed a HFD.

Gastric tension receptor mechanosensitivity of TRPV1+/+ and-/- mice fed a HFD is comparable (Fig 6A). There was no difference in the rate of gastric emptying of a solid meal in TRPV1+/+ and-/- mice fed a HFD (Fig 6B). However, the rate of gastric emptying was significantly delayed in the mice fed a HFD (Fig 6D) compared to mice fed a SLD (Fig 5B; p<0.001 for both TRPV1+/+ and-/- mice; data not illustrated on the same graph). There was no difference in the daily food intake (Fig 6C) or the final stomach content (Fig 6D) between TRPV1+/+ and-/- mice fed a HFD. On the first day of the diet, food intake in the HFD groups was significantly higher than SLD groups (TRPV1+/+: SLD, 3.24±0.22g; HFD, 4.27±0.17g (p<0.05); TRPV1-/-: SLD, 3.36±0.16g; HFD, 4.97±0.17g (p<0.05)). This was followed by a reduction in food intake up to the 2 week time point, in the HFD group, and then a gradual increase in food intake over the remaining diet period (Fig 6C).

## Discussion

These data provide the first evidence that the reduced activity of gastric vagal afferents to distension in HFD conditions is likely due to disruption of TRPV1 channel signalling. The reduction in gastric tension receptor mechanosensitivity in HFD-induced obesity was not observed in TRPV1-/- mice. In addition, pharmacological studies revealed that the TRPV1 agonist OLDA potentiates responses of gastric tension receptors to circular tension in mice fed standard mouse chow but not in mice fed a HFD. The mechanosensitivity of gastric mucosal receptors was unaffected by HFD-induced obesity and/or TRPV1 channel disruption.

Satiety signals from the stomach and small intestine, mediated by the vagus, are reduced in HFD conditions [12, 20]. In addition, consistent with previous reports on colonic [21], jejunal [22] and gastro-oesophageal vagal afferent mechanosensitivity [15], the response of gastric tension receptors to stretch are also reduced in TRPV1-/- mice. However, there was no further reduction in gastric tension receptor mechanosensitivity in TRPV1-/- mice fed a HFD. This suggests that the reduction in mechanosensitivity, observed in HFD mice in this study and by others [12, 20], may be due to disruption of the TRPV1 signalling pathway. This is supported by the observation that OLDA, a TRPV1 agonist, potentiated the response of gastric tension receptors to stretch in mice fed a SLD but not in mice fed a HFD. An alternative hypothesis is that TRPV1-/- or HFD feeding reduces gastric tension receptor mechanosensitivity to its lowest point so that both interventions fail to have an additive effect. However, we know that we can further reduce gastric tension receptor mechanosensitivity in HFD mice by exposing the afferent endings to leptin [23] and thus these afferents are capable of further reduction. In this study, only the maximum response to stretch was reduced in TRPV1-/- mice, and not the threshold. In contrast, a previous report [15] indicated the threshold for activation of gastroesophageal vagal afferents is increased in TRPV1-/-, with no change in maximum response. Therefore, a more detailed evaluation of the mechanisms behind TRPV1 effects on gastric vagal afferents is required. In adipose tissue expression of TRPV1 mRNA and protein is reduced in obesity [5], however, in the whole nodose ganglia TRPV1 mRNA content was not significantly altered in HFD mice. Thus the change in mechanosensitivity in HFD conditions cannot be simply explained by changes in TPRV1 channel expression. It should be noted, in the current study TRPV1 mRNA content was determined in the whole nodose ganglia and this may not reflect receptor expression in specific vagal afferent populations. Gastric afferents represent only about 10% of neurones in the nodose ganglia [24] and therefore it is feasible that changes in TRPV1 channels within select subpopulations of gastric afferents may not be apparent especially considering that about half of the cells within the whole nodose ganglia are TRPV1 positive [25]. In addition to direct gating mechanisms TRP channels are known to be modulated downstream of G protein-coupled receptors via a variety of second messenger pathways [26, 27]. Thus in neurons TRP channels can serve as cellular sensors to a variety of chemical stimuli. For example, endocannabinoids can modulate the activity of TRPV1 channels either directly or via G protein-coupled cannabinoid receptors [28]. In GI vagal afferent neurons this modulation of TRPV1 channels may play an important role in vagal afferent satiety signalling. In HFD conditions, where the circulating levels of appetite regulating hormones are known to change considerably [29–31], it is likely that the modulation of TRPV1 channels is altered. An understanding of how TRPV1 channels, in gastric vagal afferents, are modulated and how these interactions change in HFD induced obesity may lead to more targeted treatments of obesity. An anatomical study, by Ward *et al*., indicates that only a proportion of gastric vagal afferent endings are TRPV1 positive [32]. Therefore, the observed effect of global TRPV1 knockout on gastric vagal afferents may be secondary to knockout of TRPV1 channels on gastric epithelial or intrinsic mesenteric nerves [32, 33] potentially involved in modulation of gastric vagal afferent mechanosensitivity. This requires further investigation.

In the stomach the role of TRPV1 in gastric vagal afferent mechanosensitivity appears to be specific to gastric tension receptors as the mechanosensitivity of gastric mucosal receptors was unaffected by TRPV1 deletion. Determination of gastric mucosal receptor TRPV1 mRNA content remains to be established. However, considering the lack of effect of disrupting TRPV1 on mucosal receptor mechanosensitivity, perhaps it is not surprising that there is no change in gastric mucosal receptor mechanosensitivity in HFD conditions observed previously [12] and confirmed in this study.

Consistent with previous reports [12, 20, 34, 35] the mice fed a HFD gained more weight than the mice fed a SLD. The effect of disrupting TRPV1 channels on weight gain appears to depend on both the quantity and type of fat in the diet. In response to a diet with 26% energy from fat (lard based diet), TRPV1-/- mice gained less weight than their wild type counterparts [36]. However, in another study [34] and the current study TRPV1+/+ and-/- mice gained an equivalent amount of weight on a diet containing ~ 60% energy from fat (lard based diet). Further, a recent study reports TRPV1-/- mice on a 55% energy from fat diet (vegetable fat based diet) gain more weight than TRPV1+/+ mice [37]. Mice fed a diet with 49% energy from fat (ingredients unreported) and treated with the TRPV1 agonist capsaicin were found to be resistant to obesity [5]. In all of these studies there were no differences in weight gain between TRPV1+/+ and-/- mice fed a SLD, although one study has reported a lesser weight gain in TRPV1-/- mice fed a SLD [38]. TRPV1 channels are increasingly implicated in various metabolic processes or syndromes, related to high saturated fat intake, such as adipogenesis, lipid metabolism and type 2 diabetes [39], therefore, the observed changes in body weight under specific dietary conditions may be a result of a multitude of TRPV1 mediated metabolic effects.

The reduction in mechanosensitivity of gastric tension receptors in TRPV1-/- mice was associated with an increase in daily food intake in mice fed a SLD. From the data obtained it is difficult to determine whether this is due to an increase in meal size or the number of meals. The stomach content, measured at the end of the diet period, provides a snapshot of food intake with the limitation that the time between the meal and the measurement of food content is unknown. In TRPV1-/- mice fed a SLD stomach content was significantly greater than TRPV1+/+ mice suggesting meal size was increased. Although this is consistent with a role for the vagus in the short-term regulation of food intake and meal termination[40], it is recognised that the association between reduced mechanosensitivity of gastric vagal afferents and food intake does not imply a direct cause and effect relationship and further investigation is required. Although little is known about the role of TRPV1 channels in the nucleus of the solitary tract [41, 42] and the dorsal motor nucleus of the vagus (DMV) [43] in terms of food intake there is some evidence for a possible interaction between TRPV1 channels and leptin signalling [44] in the DMV. Therefore the observed effects on food intake in TRPV1-/- mice in the current study could be due to disruption of TRPV1 channels in central regions associated with food intake. Further studies, for example, using mice with a conditional knockout of TRPV1 channels in vagal afferents, are required. In addition to food intake gastric emptying rate was also measured. In TRPV1 +/+ and-/- mice fed a SLD there appears to be a slight delay in gastric emptying, however, this did not reach significance and thus the increase in stomach content was not due to alterations in the rate of gastric emptying further supporting the hypothesis that meal size is increased. The observation that a reduction in gastric tension receptor mechanosensitivity does not result in reduced gastric emptying is unpredicted and highlights the current limited knowledge of the physiological role of specific subtypes of vagal afferent. An alternative hypothesis for reduced gastric vagal afferent mechanosensitivity in TRPV1-/- mice is that increased food intake stretches the stomach which subsequently leads to decreased sensitivity of the gastric tension sensitive afferents. A more detailed analysis of stomach compliance and gastric vagal afferent mechanosensitivity in TRPV1-/- mice needs to be undertaken to determine the importance of this alternate hypothesis. Although there was an increase in daily food intake in TRPV1-/- mice, in line with the reduction in gastric vagal afferent mechanosensitivity, there was no associated increase in body mass compared to TRPV1+/+ mice. This discrepancy is possibly due to the increased energy expenditure necessary for the hyperactivity observed in young TRPV1-/- mice [45]. Consistent with the lack of difference, between TRPV1+/+ and-/- mice fed a HFD, in gastric tension receptor mechanosensitivity there was no difference in food intake over the 20 week diet period or stomach content at the end of the diet period. In the first day of the HFD, presumably due to the high palatability of the diet, there was a significant increase in food intake. This was followed by a dramatic drop in food intake possibly due to the satiety effects of fat mediated in the small intestine [46]. There was no difference in the rate of gastric emptying between HFD fed TRPV1+/+ and-/- mice, however, the rate of gastric emptying was significantly delayed compared to the SLD mice. Again, this is probably due to dietary fat induced small intestinal gut peptide inhibition of gastric motility [47, 48].

Previous studies have shown that the TRPV1 agonist capsaicin can acutely sensitize humans to gastric distension [49] and reduces food intake [50]. In addition, the lipid messenger oleoylethanolamide reduces short term food intake in a TRPV1-dependent manner [51]. The observation, albeit in mice, that TRPV1 channels modulate gastric vagal afferent tension receptor mechanosensitivity and may mediate the reduction in gastric vagal afferent mechanosensitivity in response to HFD induced obesity suggests that modulation of TRPV1 channels may be an important target for the regulation of food intake.
